# Proteinaceous Secretory Metabolites of Probiotic Human Commensal *Enterococcus hirae* 20c, *E. faecium* 12a and L12b as Antiproliferative Agents Against Cancer Cell Lines

**DOI:** 10.3389/fmicb.2018.00948

**Published:** 2018-05-15

**Authors:** Preeti Sharma, Sumanpreet Kaur, Raminderjit Kaur, Manpreet Kaur, Sukhraj Kaur

**Affiliations:** ^1^Department of Microbiology, Guru Nanak Dev University, Amritsar, India; ^2^Department of Molecular Biology and Biochemistry, Guru Nanak Dev University, Amritsar, India; ^3^Department of Human Genetics, Guru Nanak Dev University, Amritsar, India

**Keywords:** probiotics, vaginal *Enterococcus*, anti-proliferative, anticancer, lactic acid bacteria

## Abstract

Cancer is the second leading cause of death worldwide and its incidence is expected to grow by almost 70% in the coming 2 decades. Recent microbiome studies in cancer mice models have shown that certain commensal bacteria play protective roles against cancer. Thus, the use of commensal microflora having anticancer activities for the treatment of cancer appears to be an attractive alternative therapeutic strategy. Lactic acid bacteria (LAB) form an integral component of commensal microflora in healthy individuals. As the vaginal ecosystem is enriched in LAB genera, we screened the vaginal LAB microflora of healthy women for their anti-proliferative abilities against various human cancer cell lines. The secreted metabolites of three enterococcal strains, *Enterococcus hirae* 20c, *Enterococcus faecium* 12a and L12b, out of 92 LAB isolates selectively inhibited the *in vitro* proliferation of various human cancer cell lines in a dose-dependent manner but had no activity against normal human peripheral blood monocytes. Further, proteinase K-treatment of the cell-free supernatant (CS) of all the three enterococci abrogated their anti-proliferative abilities, thereby showing the proteinaceous nature of the secreted metabolites in the CS. The microscopic examination of the cell lines showed that CS-treatment induced apoptosis-like morphological changes in the cancer cells. Further, the probiotic characters of the strains were studied, which showed that all the three strains had broad spectrum antimicrobial activities against various Gram-positive and Gram-negative pathogens, including *Mycobacterium smegmatis*. All the strains tolerated the gastric acidity and bile juice treatments, and had strong adhesive abilities to the colonic epithelial cell line HCT-15. Furthermore, none of the strains had any known secreted virulence factors or harbored virulence genes. This preliminary study highlights an important functional role of the commensal probiotic enterococcal strains *E. hirae* and *E. faecium* for the first time by demonstrating their anticancer properties that should be further tested in the *in vivo* mammalian models.

## Introduction

Cancer has emerged as a leading cause of morbidity and mortality worldwide. In 2012, an estimated 14 million new cases of cancer were reported globally (Ferlay et al., 2015) and WHO (2017) predicted that in the coming 2 decades the number of cancer cases will rise by 70%. The number of cancer deaths stand at 8.8 million in 2015, making it the second leading cause of deaths worldwide. The rate of deaths in various types of cancer is in the order lung > liver > colorectal > stomach (WHO, 2017). Despite several therapeutic strategies available, the treatment of cancer still remains a formidable task. Chemotherapeutic drugs employed for the cancer treatment present considerable challenge due to their potential side-effects owing to their non-specificity toward normal cells. Moreover, in due course of treatment, cancer cells are known to develop resistance to the chemotherapeutic drugs. Therefore, there is an immense need for meticulous efforts to develop novel and safe anticancer agents. Recent progress in understanding the link between cancer and microbiome profiles of patients suggest that interventions that change the composition of the microbiome may affect the process of oncogenesis (Roy and Trinchieri, 2017; Zitvogel et al., 2017). Thus, the future anticancer therapies may combine the use of commensal-derived microorganisms or microbial products for the treatment of cancer (Zitvogel et al., 2017).

Probiotics are “live microorganisms which when administered in adequate amounts confer health benefits to the host” (FAO/WHO, 2002). Anticancer properties of few lactic acid bacteria (LAB) such as *Lactobacillus* (Murosaki et al., 2000; Gamallat et al., 2016) and *Pediococcus* (Dubey et al., 2016) are known. The various mechanisms through which probiotic LAB strains exert anticancer activities are by immunomodulation (Rafter, 2002), production of anticancer metabolites such as short chain fatty acids (Scheppach et al., 1995), bacteriocins (Kaur and Kaur, 2015), and the regulation of cell differentiation and apoptosis (Zhong et al., 2014). LAB probiotics are mostly shown to inhibit the growth of cancer cells indirectly through enhancing the adaptive immunity by inducing cytotoxic T cells (Lenoir et al., 2016), NK cells (Takagi et al., 2001), or cytokines (Seow et al., 2008). The direct effect of the secretory components of the LAB strains on inducing apoptosis of cancer cells is not much known. The probiotic strains that secrete anticancer metabolites may have additional advantage along with immunomodulatory properties and thus might be better putative anticancer agents.

*Enterococcus* is an important LAB genera that forms an integral part of healthy human microbiota of the gut (Noble, 1978), vagina (Brown et al., 2007), and oral cavity (Sedgley et al., 2004). Enterococci are among the earliest colonizers of the human gut (Houghteling and Walker, 2015) in the newborns. Several enterococcal strains have shown health benefits in the human clinical trials conducted for diseases such as diarrhea, irritable bowel syndrome, obesity, allergy, etc. (Franz et al., 2011). Few studies have shown the anticancer properties of enterococcal strains of food origin both *in vitro* and *in vivo* (Castro et al., 2010; Nami et al., 2015). However, not much is known about the anticancer activities of human-derived enterococcal strains. Human-derived microbial strains are known to have superior epithelial cell adhesion properties (Duary et al., 2011) and probiotic characters and therefore are successfully able to colonize the human intestinal tract (Dunne et al., 2001). Thus, the aim of the current study was to screen the cell-free supernatant (CS) of the probiotic LAB isolates from the vaginal tract of healthy women for their anti-proliferative potential against different cancer cell lines. Further, the nature and mode of action of the anti-proliferative agent in the secreted metabolites was also studied.

## Materials and Methods

### Bacterial Isolates and Physico-Chemical Characterization

The LAB were isolated from the vaginal swab samples collected aseptically from the lateral vaginal wall of 40 healthy women visiting gynecology out-patient department of Lal hospital, Amritsar, India, after taking their written informed consents. The study was approved by the Institutional Human Ethics Committee. Vaginal swabs were immersed in tubes containing sterile thioglycollate broth (HiMedia Laboratories Private Limited, Mumbai) and transported to the laboratory. Broth was incubated at 37°C and 5% carbon dioxide (CO_2_)-containing atmosphere in the CO_2_ incubator (Astec Co. Ltd., Japan) under stationary conditions for 4 h and 10-fold serial dilutions of the samples were spread onto De Man, Rogosa, and Sharpe (MRS, HiMedia) agar-medium. The MRS plates were incubated at 37°C; 5% CO_2_ atmosphere. Bacterial colonies with different morphologies were selected and preserved in 20% (v/v) glycerol-containing MRS broth at -80°C. The LAB were identified by their abilities to grow on the selective MRS media, Gram-positive staining, and catalase-negative phenotype as described in the second edition of Bergey’s manual (Holt et al., 1994; Day et al., 2001). A total of 92 LAB strains were isolated, out of which 23 isolates were identified as lactobacilli and the rest were Gram-positive cocci. Further, to characterize the genera of selected cocci isolates, physico-chemical tests such as growth at temperatures 10°C and 45°C, in MRS containing 6.5% NaCl, production of gas and hydrolysis of bile esculin disks were done (Devriese et al., 2006). To obtain fresh cultures from the frozen stocks, strains were propagated twice in MRS medium at 37°C before the experiments.

Various pathogenic bacterial indicator strains used in this study were procured from Microbial Type Culture Collection (MTCC), Institute of Microbial Technology, Chandigarh, India. The pathogens used were *Staphylococcus aureus* subsp. *aureus* MTCC 96, *Escherichia coli* MTCC 119, *Pseudomonas aeruginosa* MTCC 741, *Klebsiella pnueumoniae* subsp. *pneumoniae* MTCC 1704, *Shigella flexneri* MTCC 1457, *Vibrio cholerae* MTCC 3906, *Salmonella enterica* Typhimurium MTCC 733, *Listeria monocytogenes* MTCC 657, and *Mycobacterium smegmatis* MTCC 6. All the pathogenic bacterial strains, except *M. smegmatis* were grown and maintained on Brain Heart Infusion (BHI; HiMedia) medium at 37°C. *M. smegmatis* was grown aerobically at 37°C in 7H9 broth (HiMedia) supplemented with Middlebrook OADC growth supplement (HiMedia) containing bovine serum albumin fraction V and Tween-80.

Lactobacilli isolates (Kaur et al., 2018) used as indicator cultures were isolated from stool samples of healthy children and cultured in MRS medium at 37°C in anaerobic jars.

### Molecular Identification of the Enterococcal Isolates by 16S rDNA Sequencing

The genomic DNA of the three enterococcal strains was isolated according to the method described by Moore et al. (2004). Following DNA isolation, 16S rDNA was amplified by PCR using universal primers 27F Forward: 5′-AGAGTTGATCCTGGCTCAG-3′ and 1492P Reverse: 5′-TACGGCTACCTTGTTACGACTT-3′. DNA amplification was carried out in 0.2 mL PCR tubes by using mastercycler personal (Eppendorf, Hamburg, Germany). The PCR reaction mixture (50 μL) consisted of 25 μL of 2X PCR Master Mix (3B BlackBio Biotech India Ltd., India), 1 μL of each primer (Bioserve Biotechnologies Pvt. Ltd., India), 5 μL of template DNA, and 18 μL of nuclease free water. For PCR reaction, the initial denaturation of DNA was carried for 4 min at 95°C followed by 32 cycles of amplification. The amplification cycle consisted of denaturation step at 95°C for 1 min, primer-annealing at 56°C for 1 min 30 s, and extension at 72°C for 1 min. Reactions were completed with 10 min elongation at 72°C followed by cooling to 4°C. The PCR products were analyzed by electrophoresis on a 1.5% agarose gel at 100 V for 45 min against 100 bp step ladder. The bands were visualized with bioimaging system (GeneGenius Imaging System, Syngene Bioimaging Pvt., Ltd., India). The isolates were identified by using partial sequencing of 16S rDNA. The sequences were aligned using BLAST (NCBI) version 2 (Altschul et al., 1990). All 16S rDNA sequences were submitted to NCBI GenBank with submission numbers SUB2507370 for 12a, SUB2507390 for 20c, and SUB2507388 for L12b.

### Antimicrobial Activity of CS of Enterococci

Antimicrobial potential of the CS of 92 vaginal LAB isolates was determined by agar-well diffusion method against various pathogenic indicator strains and *Lactobacillus* isolates. To prepare CS, LAB isolates were grown overnight at 37°C in MRS media in anaerobic jar. The overnight grown cultures were centrifuged at 800 × *g* for 10 min at 4°C to obtain CS, passed through 0.22-μm syringe filters and stored at 4°C till further use.

For performing agar-well diffusion assay, the protocol by Geis et al. (1983) was followed. Briefly, the indicator strains in the log phase were spread onto their respective agar media plates and the wells with 6 mm diameter were made in the inoculated plates by using a sterilized borer. Thereafter, 100 μL of CS of the LAB isolates were added to the wells and the plates were incubated at 4°C overnight to allow the CS to diffuse. The plates were then incubated at 37°C under aerobic conditions for the growth of pathogenic strains and in anaerobic jars for the growth of fecal lactobacilli strains. The zones of inhibition were measured in mm after 24 h. The strains having broad spectrum antimicrobial activity against both Gram-positive and Gram-negative pathogens were selected for further studies.

### Probiotic Properties of Enterococcal Strains

#### Gastric and Bile Juice Tolerance

Cells from overnight culture of enterococcal isolates were harvested by centrifugation at 800 × *g* for 10 min at 4°C and washed three times with 0.1 M phosphate buffered saline (PBS; pH 7.2). The cell pellet was suspended in simulated gastric juice (SGJ) at the concentration of 1 × 10^8^ CFU/mL and incubated at 37°C with 5% CO_2_. SGJ was composed of 3.2 g/L pepsin and 2 g NaCl/L, and the pH was adjusted to 2.0 with sterile 5N HCl (Huang and Adams, 2004). Cells suspended in PBS were used as controls. Survival cell counts were determined at different time points (0, 2, and 4 h) by plating onto MRS agar plates and incubating at 37°C with 5% CO_2_.

Bile salt tolerance of enterococcal isolates was determined by using method of Pereira and Gibson (2002) with some modifications. MRS broth with 0.3% (w/v) oxgall and without oxgall was inoculated with 1 × 10^8^ CFU/mL of overnight grown cultures and incubated at 37°C; 5% CO_2_. Survival cell counts were determined at different time points (0, 2, and 4 h) by plating onto MRS agar plates and incubating at 37°C with 5% CO_2_. Both the experiments were performed three times in triplicates.

#### Biofilm Formation

Biofilm formation potential of the enterococcal isolates was estimated by crystal violet assay (Stepanović et al., 2000). The potential of enterococci to form biofilm was tested at 3 different pH values (4, 5, and 6) for different time points (24, 48, and 72 h). The optical density (OD) at wavelength 595 of overnight grown cultures was adjusted to 0.2 and 15 μL was added to the wells of 96-well plate containing 135 μL MRS broth. Plates were incubated at 37°C with 5% CO_2_ for different time periods to allow the formation of biofilms. After incubation period, plates were washed thrice with 100 μL sterile PBS to remove non-adherent cells. The adhered biofilm was fixed with methanol and stained with 2% (w/v) crystal violet. The wells were washed thrice with PBS to remove excess stain. The stain was released from the biofilms with 160 μL of 33% (v/v) glacial acetic acid and absorbance of the wells was determined at wavelength 595 nm. Uninoculated MRS broth served as control. Three independent experiments were performed in triplicates. Based on the absorbance, the strains were categorized as non-biofilm producers if OD ≤ OD_C_, weak biofilm producers if OD_C_ < OD ≤ 2OD_C_, moderate biofilm producers = 2OD_C_ < OD ≤ 4OD_C_, strong biofilm producers = 4OD_C_ < OD, where OD = OD of inoculated well and OD_C_ = OD of control well.

#### Adhesion to Intestinal Cells

Adhesion of the enterococcal isolates was assayed on the monolayers of HCT-15 cells grown on cover slips in 60 mm petridishes. HCT-15 monolayers were washed twice with PBS and 3 mL of DMEM containing enterococcal cells in the ratio 1:100 was added and incubated for 1 h at 37°C; 5% CO_2_. Following incubation, all of the dishes were washed four times with PBS to release unbound bacteria. The cells were then fixed with methanol for 10 min and stained with Giemsa stain solution for 30 min. The dishes were washed with PBS until no color was observed, dried, and observed under bright field microscope. Each adhesion assay was performed in triplicates with cells from three successive passages (8–13 cell passages). The adherent enterococci in five random microscopic fields were counted for each test. Bacterial strains were scored as non-adhesive when fewer than 40 bacteria were present in 5 fields, adhesive when 41–100 bacteria were present in 5 fields, and strongly adhesive when more than 100 bacteria occurred in 5 fields (Fernández et al., 2003).

### Safety Determinants

#### Hemolytic, Gelatinase, and Casein Hydrolase Activity

To evaluate hemolytic activity, enterococcal isolates were streaked on Columbia agar plates containing 5% (v/v) sheep blood (HiMedia) and incubated at 37°C for 24 h. Hemolytic activity was detected by appearance of halo around the colonies: green zone for α-hemolysis, clear zone for β-hemolysis and no halo indicated γ-hemolysis (Barbosa et al., 2010). To evaluate the proteolytic activity of the isolates against gelatin and casein, BHI media was supplemented with 1% gelatin (Kanemitsu et al., 2001) and 1.5% skim milk (Lisiecki, 2017), respectively. Cultures were streaked on the BHI plates and incubated at 37°C for 24 h. A halo around the colonies indicated a positive result for gelatinase and casein hydrolase production.

#### PCR Amplification of the Virulence Determinant Genes

*Enterococcus* isolates were tested for the presence of genes encoding various virulence factors by using PCR amplification method. The amplification conditions and primers used are mentioned in **Table 1**. Total chromosomal DNA from overnight grown cultures of the isolates was extracted according to the method by Moore et al. (2004). DNA was quantified by using 1% agarose gel stained with ethidium bromide. For the detection of virulence genes, PCR was performed in 50 μL reaction mixture containing 5 μL bacterial DNA template (50 ng), 25 μL of 2X PCR Mastermix, 1 μL of each primer (Bioserve Biotechnologies), and 18 μL of nuclease free water by using mastercycler personal (Eppendorf). The PCR reaction conditions used were DNA denaturation at 95°C for 4 min. followed by 32 cycles of amplification. The amplification cycle consisted of denaturation step at 95°C for 1 min, annealing temperature as mentioned in **Table 1** followed by extension at 72°C for 1 min. Reactions were completed with 10 min elongation at 72°C followed by cooling to 4°C. PCR products were analyzed in 1.5% agarose gel stained with ethidium bromide and visualized by using bioimaging system.

**Table 1:** PCR primers and reaction conditions used for the detection of genes implicated in virulence of *enterococcal* isolates.

Virulence determinant genes	Primer sequence (5′-3′)	Amplicon (bp)	Annealing temperature (°C)	Reference
*agg* (aggregation substance)	F:AAGAAAAAGAAGTAGACCAAC R:AAACGGCAAGACAAGTAAATA	1553	50	Eaton and Gasson, 2001
*esp* (Enterococcal surface protein)	F:AGATTTCATCTTTGATTCTTGG R:AATTGATTCTTTAGCATCTGG	510	48	Vankerckhoven et al., 2004
*gel E* (extracellular metallo-endopeptidase)	F:ACCCCGTATCATTGGTTT R: ACGCATTGCTTTTCCATC	419	51	Sabia et al., 2008
*cyl* (cytolysin)	F: ACTCGGGGATTGATAGGC R: GCTGCTAAAGCTGCGCTT	688	58	Vankerckhoven et al., 2004


#### Antibiotic Susceptibility

Antibiotic susceptibility of the isolates was determined by Kirby Bauer disk diffusion method (Bauer et al., 1966) in MRS agar media. Isolates were grown overnight in MRS broth at 37°C. A total of 100 μL of the culture having 1 × 10^6^ CFU/mL was spread plated onto MRS agar-containing plates. Antibiotic disks (HiMedia) were placed on the MRS agar plates and incubated at 37°C for 24 h at 5% CO_2_. Diameters of the zone of inhibition around the disks were measured in mm. The results were reported as sensitive, intermediate or resistant to various antibiotics according to the breaking points recommended by the Clinical and Laboratory Standards Institute (CLSI, 2012) guidelines for enterococci.

### Assessment of Anti-proliferative Activity of CS

#### Cancer Cell Lines and Preparation of Peripheral Blood Mononuclear Cells (PBMC)

The various human cancer cell lines used in the study were cervical cell line, HeLa; lung carcinoma, A549 and colonic epithelial cell line, HCT-15. These cell lines were procured from National Cell Center, Pune, India. The cell lines were cultured in Dulbecco’s Modified Eagle Medium (DMEM; Sigma-Aldrich, United States) supplemented with 10% fetal bovine serum (Sigma-Aldrich, United States) in tissue culture flasks at 5% CO_2_-containing atmosphere and at 37°C. These cells were collected in the confluent phase by treating with Trypsin–Hank’s Balanced Salt Solution. The cells suspension was centrifuged at 120 × *g* for 15 min. The supernatant was removed and 5 mL of DMEM was added to the cell pellet. The cell number was counted using hemocytometer.

The PBMC were isolated from the whole blood by density gradient centrifugation. For this, 5 mL of peripheral blood was withdrawn from the antecubital vein and transferred to ethylenediaminetetraaceticacid-coated vial. The blood was then diluted with PBS in the ratio of 1:1 and 5 mL of HiSep (HiMedia) was added in a centrifuge tube. The diluted blood was poured onto HiSep solution along the wall in the ratio of 1:2 and centrifuged at 128 × *g* for 20 min. The white ring of PBMC was harvested carefully, washed thrice with PBS, and suspended in DMEM. The cell number was counted using hemocytometer.

#### MTT [3-(4,5-dimethylthiazol-2-yl)-2,5-diphenyltetrazolium Bromide] Assay

The MTT assay (Mosmann, 1983) was used to determine the anti-proliferative activities of the CS of the enterococcal isolates against cancer cell lines HeLa, HCT-15, and A549 and normal human cells, PBMC. CS was prepared by centrifuging overnight grown culture of the enterococcal isolates at 800 × *g* for 10 min. The pH of CS was set at 7.2 with 1N NaOH and CS was lyophilized and diluted in DMEM media to obtain desired concentrations of 1.5–50 μg/mL. The solution was filtered sterilized through 0.22-μm syringe filters (Milipore, United States) and used for the MTT assay.

For setting up the MTT assay, 100 μL of the cell suspension containing 4 × 10^5^ cells/mL was added to each well of the 96-well plate and the plate was kept in the CO_2_ incubator with 5% CO_2_, at 37°C for 24 h. After 24 h, 100 μL of the serially diluted CS or lyophilized MRS (as negative control) in DMEM was added to the wells. The plate was again maintained in the CO_2_ incubator for 24 h. The supernatant was discarded and 100 μL of MTT (5 mg/mL for PBMC and 0.5 mg/mL for cell lines) was added to each well and the plate was incubated in CO_2_ incubator at 37°C for another 4 h. After incubation, medium containing MTT was discarded and 100 μL of dimethyl sulfoxide (HiMedia, India) was added to the wells to dissolve the blue formazan crystals. The OD was read at wavelength 570 nm on the microplate reader (Multiskan^®^ EX by LabSystems, Finland). The proliferation of cells under treatment was assessed according to the following formula:

$$\text{Percentage of proliferation = }\frac{\text{Absorbance of CS-treated wells}}{\text{Absorbance of MRS-treated control wells}}\times 100$$

To determine the proteinaceous nature of the component of CS responsible for the anti-proliferative activity, the CS was subjected to treatment with proteinase K (1 mg/mL; HiMedia) for 2 h at 37°C, followed by deactivation of proteinase K by heating at 60°C for 15 min. The residual anti-proliferative activity of the CS was determined by using MTT assay as described previously. The untreated-CS was used as positive control and MRS was used as negative control.

#### Apoptosis Detection by Staining Techniques

Apoptosis of eukaryotic cells is associated with certain morphological changes in their cell membranes and DNA that could be studied microscopically (Elmore, 2007). To study the effect of CS on the morphology of HCT-15 cells, they were treated with 50 μg/mL of CS and then stained with dyes, Hoechst 33342, propidium iodide (PI), and Giemsa (Merck, Darmstadt, Germany). Sterile coverslips were placed in wells of 6-well tissue culture plate (CoStar, United States). HCT-15 cells were added to each well of the plate at a concentration of 1.2 × 10^6^ cells/well in DMEM and incubated at 37°C in the CO_2_ incubator. After 24 h, CS of enterococcal isolates and MRS as controls were added to the wells and the plate was again incubated at 37°C in the CO_2_ incubator for 24 h. The cells were fixed with 4% paraformaldehyde for 5 min and washed thrice with PBS before staining with Hoechst 33342 (1 μg/mL) and PI (5 μg/mL) for 15 min, and with Giemsa (1:9) for 20 min. After staining, the coverslips were washed thrice with PBS to remove excess stain. Hoechst 33342 and PI-stained coverslips were viewed under fluorescent microscope and Giemsa-stained coverslips were viewed under bright field microscope (Nikon A1R, Japan).

### Statistical Analysis

Data were analyzed by one-way ANOVA. Significant differences of means (*p* < 0.05) were compared through independent Student’s *t-*test by using SPSS 17.0.

## Results

### Antimicrobial Activity

Out of the 92 tested vaginal LAB isolates, 3 cocci, 12a, 20c, and L12b had broad spectrum antimicrobial activities in CS against both Gram-positive and Gram-negative pathogens. The CS of 12a and 20c inhibited the growth of *S. enterica* Typhimurium, *E. coli*, *S. flexneri*, *M. smegmatis*, *V. cholerae*, and *L. monocytogenes*, whereas, CS of L12b inhibited *S. enterica* Typhimurium, *E. coli*, *S. flexneri*, *M. smegmatis*, *L. monocytogenes*, and *S. aureus* (**Table 2**). None of the isolate inhibited the growth of human pathogens *P. aeruginosa* and *K. pneumoniae*. Further, the antimicrobial activities of CS were also screened against 7 Lactobacilli spp. isolated from human gut. None of the CS inhibited any *Lactobacillus* isolate (**Table 2**).

**Table 2:** Antimicrobial activity of the enterococcal isolates against various indicator microorganisms.

Indicator microorganism	Susceptibility profile		
	
	12a	L12b	20c
*S. enterica* Typhimurium	S	S	S
*E. coli*	S	S	S
*S. flexneri*	S	S	S
*L. monocytogenes*	S	S	S
*V. cholera*	S	R	S
*M. smegmatis*	S	S	S
*P. aeruginosa*	R	R	R
*S. aureus*	R	S	R
*K. pneumonia*	R	R	R
*L. plantarum* L14	R	R	R
*L. fermentum* L32	R	R	R
*L. pentosus* S45	R	R	R
*Lactobacillus* spp. L13	R	R	R
*Lactobacillus* spp. L12	R	R	R
*Lactobacillus* spp. L18	R	R	R
*Lactobacillus* spp. S49	R	R	R


*The antimicrobial activities of the CS of enterococcal isolates were determined by using agar gel diffusion assay. The pH of CS was set to 6.5 by using 1 N NaOH. The experiment was performed in triplicates. S, diameter of the zone of inhibition > 10 mm. R, diameter of the zone of inhibition < 10 mm.*

Further, the zones of inhibition of CS of all the three isolates against various susceptible strains remained same with and without pH neutralization (data not shown), thereby showing that the antimicrobial effect was not due to acidic conditions of CS.

### Physico-Chemical and Genetic Characterization of the Enterococcal Isolates

Three LAB isolates 12a, L12b, and 20c selected for the study were characterized by both physico-chemical and genetic methods, and were further screened for their probiotic, virulence, and anti-proliferative properties. Physico-chemical tests showed that all the three isolates belonged to the genera *Enterococcus* as they all showed growth at temperatures 10°C and 45°C, in MRS-containing 6.5% NaCl and hydrolyzed bile esculin (data not shown). The isolates were further identified by using partial sequencing of 16S rDNA. The sequences were aligned using BLAST (NCBI) version 2. The isolates 12a and L12b with Genbank NCBI accession numbers KY785661 and KY785374, respectively, had 99% sequence similarities to *E. faecium*; while the isolate 20c (accession number KY785319) had 99% similarity to *E. hirae*.

### Probiotic Properties

#### Gastric and Bile Juice Conditions

**FIGURE 1:**
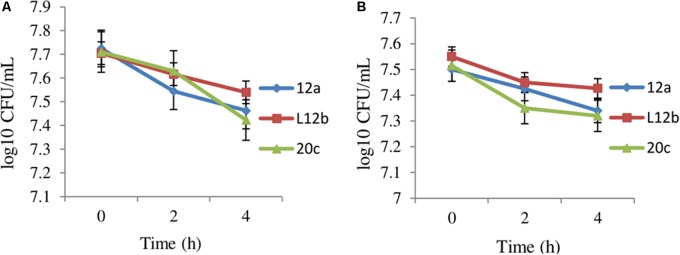
Effect of **(A)** simulated gastric juice and **(B)** bile juice treatment on the viabilities of enterococcal isolates after 4 h. Error bars are representative of SD of the three independent experiments performed in triplicates.

The oral administration of probiotics subjects them to the harsh acidic environment in the stomach and to bile juices in the small intestine. Therefore, to determine the ability of enterococcal isolates to survive in gastric juice, the survival of the isolates 12a, L12b and 20c were evaluated in SGJ having pH 2.0 and in the presence of bile salts. Both SGJ and bile treatment resulted in less than 0.5 log CFU change in the viabilities of all the three enterococcal isolates after 4 h (**Figure 1**). Isolate L12b appeared to be comparatively more resistant to the effects of both SGJ and bile treatment as compared to isolates 12a and 20c.

#### Biofilm Formation and Adherence of Enterococci to HCT-15 Cell Line

Biofilm formed by probiotic microorganisms cover epithelial cell receptors in the gut and inhibit colonization by undesirable microorganisms. Thus, biofilm formation is a desirable characteristic for probiotic cultures. In this study, we observed that the three enterococcal isolates possessed the ability to form well-structured biofilm. All the isolates formed strong biofilm at pH 6 and at 48 h. At pH 4 and 5, only isolate L12b showed strong biofilm formation at 48 h (**Table 3**).

**Table 3:** Biofilm-forming potential of the enterococcal isolates.

Enterococcal isolate	Biofilm forming potential at different pH and time periods								
	
	pH 4			pH 5			pH 6		
			
	24 h	48 h	72 h	24 h	48 h	72 h	24 h	48 h	72 h
12a	W	W	M	W	M	M	S	S	S
L12b	W	M	S	W	S	M	S	S	M
20c	W	W	W	W	M	W	M	S	S


*S: strong biofilm producers, M: moderate biofilm producers, W: weak biofilm producers. The enterococcal biofilms were formed in MRS media of different pH in 96-well microtitre plates and analyzed at different time points by using crystral violet assay. Based on OD_*59*5_ of the well, strains were classified as: weak biofilm producers (OD_*C*_ < OD ≤ 2OD_*C*_), moderate biofilm producers (2OD_*C*_ < OD ≤ 4OD_*C*_), and strong biofilm producers (4OD_*C*_ < OD), where OD = OD of inoculated well and OD_*C*_ = OD of uninoculated well. Experiments were performed three times in triplicates.*

**FIGURE 2:**
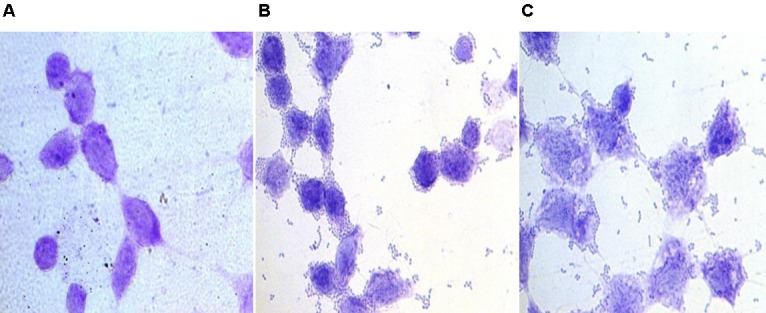
Bright field microscopic images showing adhesion of enterococcal cells to colonic epithelial cell line HCT-15. Giemsa-stained HCT-15 cells **(A)** without enterococci, **(B)** with *E. faecium* 12a, and **(C)** with *E. hirae* 20c.

Further, the adherence of the enterococcal isolates to the HCT-15 cell line was studied microscopically. All the three enterococcal isolates were strongly adhesive as 1 h incubation of bacterial cells with the cell line resulted in more than 100 bacterial cells bound to the HCT-15 cell line in the 5 fields (**Figure 2**).

### Virulence Factors

As some pathogenic strains of *Enterococcus* are known to possess virulence factors (Jett et al., 1994) that play important role in the pathogenesis of enterococcal infection, the secreted virulence factors were studied. The results showed that no gelatinase, casein hydrolase or hemolytic (γ-hemolysis) activities were observed in any of the three enterococcal cultures.

Further, the PCR amplification of virulent genes *agg*, *esp*, *gel E*, and *cyl* was negative in all the three enterococcal isolates thereby showing them as safe non-virulent strains.

### Antibiotic Susceptibility

Probiotic candidate should be screened for antibiotic resistance as they can act as potential reservoirs of transmissible antibiotic resistance genes. Antibiotic susceptibility pattern of the isolates was evaluated against various antibiotics (**Table 4**). All the three enterococcal isolates were sensitive to all the three β-lactam antibiotics and teicoplanin. Among fluoroquinolones, L12b and 12a were sensitive to both moxifloxacin and gatifloxacin. Both 20c and L12b were susceptible to tetracycline. All the three isolates were resistant to ciprofloxacin, erythromycin, and azithromycin.

**Table 4:** Antibiotic susceptibility profile of vaginal enterococcal isolates.

Antibiotic	Sensitivity profile		
	
	20c	12a	L12b
**β-lactams**			
Penicillin	S	S	S
Ampicillin	S	S	S
Carbenicillin	S	S	S
**Macrolides**			
Azithromycin	R	R	R
Erythromycin	R	R	R
**Fluoroquinolones**			
Ciprofloxacin	R	R	R
Moxifloxacin	R	S	S
Gatifloxacin	I	S	S
**Glycopeptides**			
Vancomycin	I	S	S
Teicoplanin	S	S	S
**Oxazolidinones**			
Linezolid	I	R	S
**Tetracyclines**			
Tetracycline	S	I	S


*Kirby Bauer disk diffusion method was performed, and zone of inhibition was measured in the presence of various antibiotics. Standard interpretation of antimicrobial susceptibility tests of enterococci with disk diffusion method in accordance to CLSI (2012) standards. S, susceptible; I, intermediate; R, resistant. Experiment was performed in triplicates.*

### Anti-proliferative Assay

**FIGURE 3:**
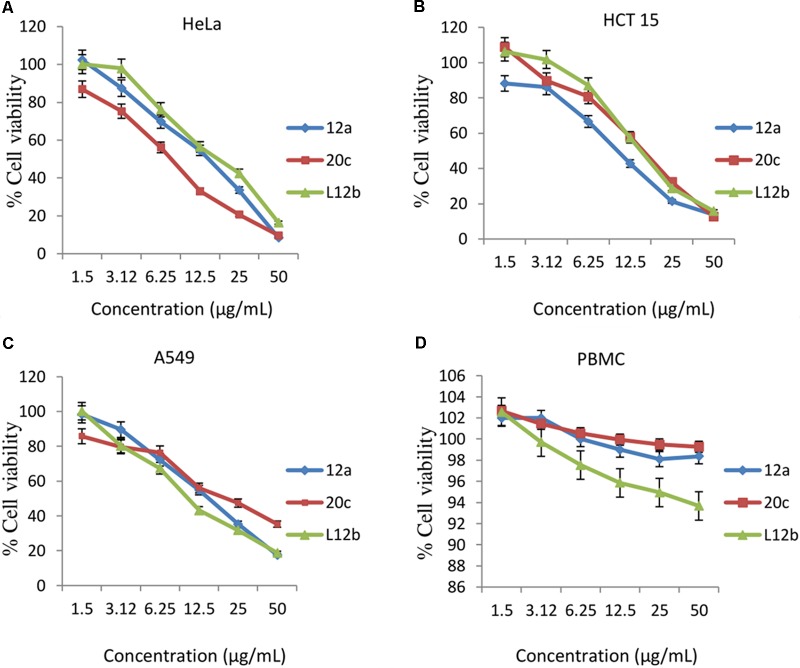
Dose-dependent effect of the secreted metabolites of the CS of enterococcal isolates on the viabilities of **(A)** HeLa, **(B)** HCT 15, **(C)** A549, and **(D)** Human PBMCs after 24 h treatment. Error bars are representative of SD of the three independent experiments performed in triplicates.

Evaluation of anti-proliferative activities of the CS of enterococcal isolates on the cancer cell lines HeLa, A549, and HCT-15 was done. CS of all the three enterococcal isolates had dose-dependent anti-proliferative activity against all the cancer cell lines tested. CS of isolate 12a, 20c, and L12b at the highest dose of 50 μg/mL reduced the viabilities of HeLa to 8.4, 9.7, and 16.4%, respectively (**Figure 3A**). The 50% inhibitory concentration (IC_50_) values of the CS of 12a, 20c, and L12b for HeLa were calculated as 12.5, 7.7, and 15.9 μg/mL, respectively.

Similarly, the secretory metabolites all the three enterococcal isolates inhibited the growth of HCT-15 (**Figure 3B**) at the maximum dose of 50 μg/mL. The viabilities of HCT-15 were reduced to 13.7, 14.4, and 15.8% after treatment with the CS of 12a, 20c, and L12b, respectively. The IC_50_ values calculated for the CS of 12a, 20c, and L12b were 9.9, 14.4, and 15.3 μg/mL, respectively.

Further, 24 h treatment of the cell line A549 with the CS of the enterococcal isolates12a, 20c, and L12b at the concentration 50 μg/mL, reduced the viabilities to 17.5, 21.3, and 18.7%, respectively (**Figure 3C**). The IC_50_ values calculated for the CS of 12a, 20c, and L12b were 14.01, 21.3, and 11.7 μg/mL, respectively.

The antiproliferative effect of CS of all the three enterococcal isolates was also determined against normal human PBMCs. The residual viabilities of PBMC after treatment with the CS of 12a, L12b, and 20c were 74.5, 76.5, and 81.5 %, respectively (**Figure 3D**).

**FIGURE 4:**
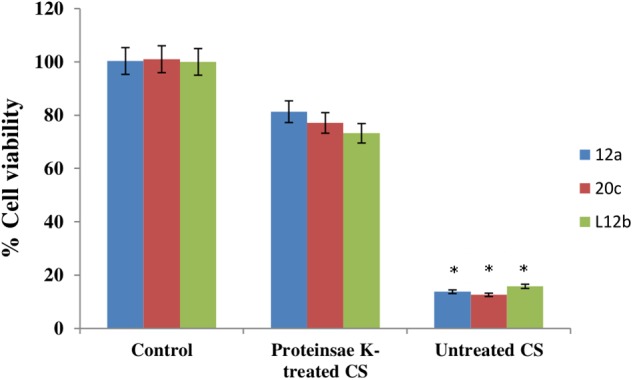
Anti-proliferative effect of CS (50 μg/mL) of enterococcal isolates, with and without proteinase K (1 μg/mL) treatment on HCT-15 cell line. Untreated HCT-15 was used as control. Error bars are representative of SD of the three independent experiments performed in triplicates. Asterisk denotes statistically (*p* < 0.001) significant difference as compared to the respective controls.

Proteinase K treatment of CS of all the three enterococcal isolates resulted in abrogation of the anti-proliferative effect of CS (**Figure 4**). The viabilities of HCT-15 were reduced only by 18.7, 22.9, and 26.8% with proteinase K-treated CS of 12a, 20c, and L12b, respectively. Thus, the anti-proliferative activity of CS of all the isolates is due to some proteinaceous substance present in the CS.

### Microscopic Detection of Morphological Changes in the CS-Treated Cell Lines

**FIGURE 5:**
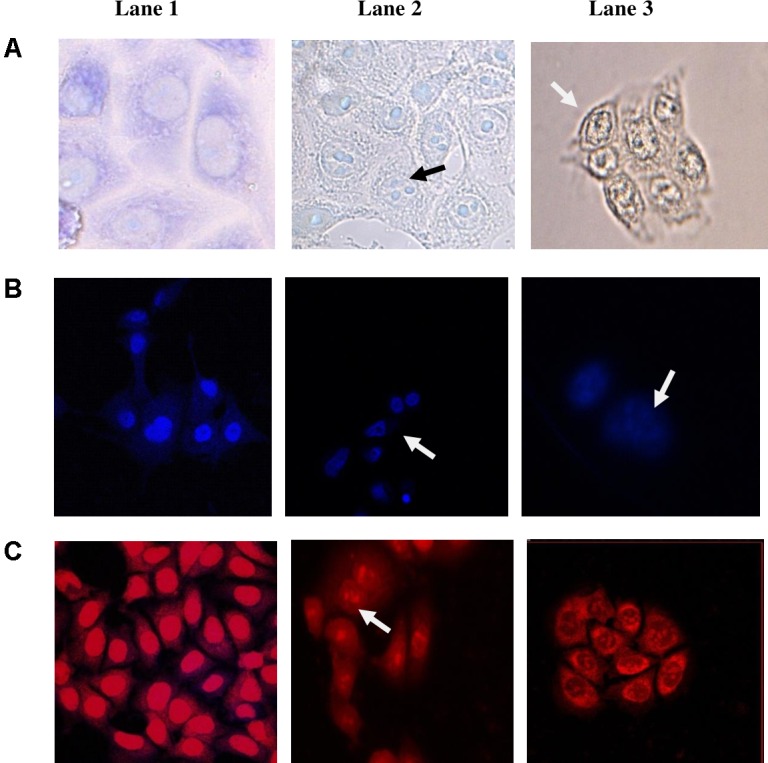
Morphological changes induced in HCT-15 following 24 h treatment with CS of enterococcal isolates. **(A)** Giemsa-stained, **(B)** Hoechst 33342-stained, and **(C)** PI-stained HCT-15 cells. Lane 1: untreated HCT-15 cells; Lane 2: HCT-15 cells treated with CS of 12a; and Lane 3: HCT-15 cells treated with CS of 20c. Arrows marked in Giemsa-stained cells show cell shrinkage along with nuclear fragmentation. Arrows marked in PI and Hoechst 33342-stained cells show nuclear condensation and fragmentation.

Further, to study the mechanism of the anti-proliferative activity of CS, the morphological changes of CS-treated cancer cell line HCT-15 cells were studied by bright field and fluorescent microscopy. Apoptosis also known as programmed cell death involves microscopically visible morphological changes such as chromatin condensation, margination, cell shrinkage, membrane blebbing, and formation of apoptotic bodies (Leite et al., 1999). Most of the cells in the control sample (treated with MRS) remained viable with normal cell morphology with clear outline of cell membrane and nucleus (**Figure 5**; Lanes A1–C1). On the other hand, the cells treated with the CS of enterococcal isolates 12a and 20c showed morphological changes typical of apoptosis (**Figure 5**). The CS-treated and Giemsa-stained cells (Lanes A2 and A3) showed cell shrinkage, chromatin condensation and nuclear fragmentation. Whereas CS-treated and fluorescent-stained (Lanes B2, B3, C2, and C3) cells showed nuclear condensation and fragmentation.

## Discussion

In this study, the selective anti-proliferative and apoptotic effects of the secreted proteinaceous metabolites of the vaginal *E. hirae* and *E. faecium* have been shown against various cell lines for the first time. Further, the strains exhibited good probiotic properties that make them promising candidates for use as prophylactic and therapeutic anticancer agents. The other two studies that reported the *in vitro* anti-proliferative effects of the secreted proteinaceous metabolites of LAB were that of *E. durans*, isolated from fermented food (Haghshenas et al., 2014) and *E. faecalis* isolated from human vagina (Nami et al., 2014b). In both the studies, the CS was shown to inhibit the human cancer cell lines HeLa, mammary cancer cell line MCF-7 and gut epithelial cell lines, AGS and HT-29. The effect of the CS of *Enterococcus* spp. on the lung carcinoma cell line A549 is reported for the first time in our study. Furthermore, the cell viability of HeLa in our study was reduced in the range from 82.6 to 91.6%; whereas in the previous studies the CS of *E. durans* induced 60% (Haghshenas et al., 2014) and *E. faecalis* induced 71.9% reduction (Nami et al., 2014b) in the viabilities of HeLa cells after 24 h at the same concentration.

The secreted metabolites from other LAB isolates such as *L. plantarum* 17C (Haghshenas et al., 2015), *L. plantarum* 5BL (Nami et al., 2014a), *Pediococcus pentosaceus* GS4 (Dubey et al., 2016), and *E. lactis* IW5 (Nami et al., 2015) were also shown to inhibit the proliferation of cancer cell lines, *in vitro*, but the type of the component in the CS responsible for the anti-proliferative activity is not known.

The proteinaceous nature of the component in the CS responsible for the anti-proliferative activities was confirmed by its proteinase K treatment, which significantly (*p* < 0.05) reduced the anti-proliferative activity against HCT-15 cell line. LAB are known to secrete cationic proteins or peptides known as bacteriocins in the CS that inhibit the growth of cancer cells (Kaur and Kaur, 2015; López-Cuellar et al., 2016). Bacteriocins are positively charged peptides that are known to selectively bind and destabilize the membranes of cancer cell lines owing to the enhanced negative charge, membrane fluidity and numbers of microvilli on the surface of cancer cells as compared to normal cells (Kaur and Kaur, 2015). Thus, the protein component of the CS that mediated anticancer activities may be a bacteriocin-like molecule or other proteins such as enzymes. The morphological changes observed microscopically suggested that the CS of the enterococcal isolates induced apoptosis in the cell line HCT-15. Other studies also reported apoptosis as the mode of action of CS on cancer cell lines (Haghshenas et al., 2014; Nami et al., 2014b). Further, as reported earlier our results also showed that CS selectively inhibited cancer cell lines and had no effect on the normal human cells (Haghshenas et al., 2014; Nami et al., 2014b).

Another indirect mechanism through which enterococci may have prophylactic action against cancer is by modulating the gut microbiota. All the three *enterococcal* isolates inhibited the growth of Gram-negative pathogens but not that of *Lactobacillus* spp. The Gram-negative microorganism-associated molecular patterns such as lipopolysaccharide have been implicated in various types of cancer (Schwabe and Jobin, 2013). Also Gram-negative bacteria such as *E. coli*, *S. enterica*, *Shigella* spp., etc., are known to produce genotoxins such as cytolethal distending toxin (Graillot et al., 2016) and colibactins (Arthur et al., 2012) that cause DNA damage and are known to promote the development of cancer. Thus, *enterococcal* isolates through inhibition of the growth of Gram-negative pathogens may protect against the development of cancer. The potential of enterococci to inhibit the *Mycobacterial* spp. has been shown for the first time in this study.

Further, the probiotic properties of enterococci were explored. Probiotics are administered orally and therefore the condition of the gastrointestinal tract is the first physiological challenge faced by them. Human stomach has a pH value ranging from 1.5 to 4.5 and it can take up to 3 h for food to get ingested (Jacobsen et al., 1999). The putative probiotic strain should be able to remain alive during transit through gastrointestinal tract at low pH and in the presence of bile salt. All the enterococcal isolates in this study were resistant to SGJ and their viabilities were not affected by more than 0.5 log CFU. These results are in concordance with the results of previous studies (Banwo et al., 2013; Nami et al., 2014b). Once inside the intestine, probiotic microorganisms must adhere to intestinal mucosa to facilitate their colonization and to prevent their removal by peristalsis. The ability for biofilm formation is associated with cell adherence potential (Boris et al., 1997). All the isolates possessed the ability to form strong biofilms at pH 6 that is similar to the physiological intestinal pH. Furthermore, adhesion to intestinal epithelial cells is an important feature for colonization and competitive exclusion of pathogens by probiotics. *In vitro* studies performed to study the adherence abilities of the isolates to the colonic epithelial-like cell line HCT-15, showed that more than 100 enterococcal cells could be counted in each field having 3–5 HCT-15 cells, thereby showing strong adherence of all the isolates to the colonic cells.

The safety features of enterococci were evaluated in terms of antibiotic profile and virulence factors. FAO/WHO (2002) addressed the problem of transmissible antibiotic resistant genes in the probiotics and recommended the evaluation of its antibiotic profile. The antibiotic susceptibility profile showed that all the isolates were sensitive to β-lactam antibiotics, which are the first line of treatment for enterococci infections. Among fluoroquinolones, L12b and 12a showed sensitivity to moxifloxacin and gatifloxacin; however, all of them were resistant to ciprofloxacin. Similarly, L12b and 12a were sensitive to vancomycin, whereas L12b and 20c were susceptible to tetracycline. Thus, the molecular mechanisms of vancomycin resistance in 20c and tetracycline resistance in 12a should be further evaluated to study their transmissible nature. All the isolates were resistant to macrolides, erythromycin, and azithromycin. Thus, based on the antibiotic susceptibility profile, L12b and 12a appear to be more sensitive to various classes of antibiotics as compared to 20c.

Safety aspect of the strains was assessed as per European Food Safety Authority (2012) guidelines both by determining PCR amplification of virulence genes such as *agg*, *gel E*, *cyl*, and *esp*, and by using bioassays to screen for the secreted virulence factors. As reported for the *E. faecium* probiotic strains, such as SF68, none of the isolates showed the presence of virulence factors and therefore all can be considered as safe probiotic candidates (Kayser, 2003).

Thus, this preliminary study explored the functional properties of human commensal enterococci. The beneficial roles of gut microflora in inhibiting tumor growth (Zitvogel et al., 2017) and modulating the therapeutic effects of anticancer drugs (Viaud et al., 2013; Roy and Trinchieri, 2017) is increasingly being realized. For example, Sivan et al. (2015) showed that the gut microflora of mice that responded to anticancer immunotherapy had the abundance of commensal *Bifidobacterium* spp. as compared to those that did not respond. Hence, the commensal *Bifidobacteria* were isolated and orally administered to melanoma mouse model that resulted in reduced tumor growth. Studies in germ-free mice have shown that gut microflora, particularly Gram-positive bacteria are required for the anticancer activity of the anticancer drug, cyclophosphamide (Viaud et al., 2013). Cyclophosphamide-treatment of mice increased the gut permeability allowing gut commensal, *E. hirae* to translocate to lymph nodes, where it induced cytotoxic T cell response to the lung and ovarian cancer (Viaud et al., 2013). In line with these studies, our results further demonstrated that commensal *Enterococcus* spp. have the ability to directly inhibit the proliferation of cancer cell lines by secreting anticancer metabolites.

## Conclusion

All the three probiotic enterococcal strains *E. hirae* 20c, *E. faecium* 12a, and L12b secreted potent proteinaceous anticancer components that selectively inhibited the human cancer cell lines but not the normal cells. Therefore, they seem to be the promising candidates that need further evaluation in *in vivo* studies for their therapeutic potential. Secondly, the protein component of the CS should be purified and characterized to determine its therapeutic efficacy both *in vitro* and *in vivo*.

## Author Contributions

SrK conceived the idea and supervised the experiments. PS and SrK designed the experiments. PS performed all of the experiments except MTT assay. SpK performed the microscopic studies, whereas RK and MK designed and performed the MTT assay. All authors discussed the results and contributed to the final manuscript.

## Conflict of Interest Statement

The authors declare that the research was conducted in the absence of any commercial or financial relationships that could be construed as a potential conflict of interest.
